# Applicability research of thermodynamic models of gas hydrate phase equilibrium based on different equations of state

**DOI:** 10.1039/d2ra00875k

**Published:** 2022-05-26

**Authors:** Geng Zhang, Jun Li, Gonghui Liu, Hongwei Yang, Honglin Huang

**Affiliations:** China University of Petroleum-Beijing Beijing 102249 China lijun446@vip.163.com; China University of Petroleum-Beijing at Karamay Karamay 834000 China

## Abstract

Choosing an appropriate equation of state and thermodynamic model is very important for predicting the phase equilibrium of a gas hydrate. This study is based on statistical thermodynamics, considering the changes in water activity caused by gas dissolution, and deriving and summarizing four thermodynamic models. Based on the 150 collected experimental data points, the accuracy of the four thermodynamic models in predicting the phase equilibrium of methane hydrate, ethane hydrate, and carbon dioxide hydrate were compared. In addition, the influence of five equations of state on each thermodynamic model's phase equilibrium prediction accuracy is compared. The analysis results show that in the temperature range of 273.40–290.15 K, the Chen–Guo model is better than other thermodynamic models in predicting the phase equilibrium of methane hydrate by using the Patel–Teja equation of state. However, in the temperature range of 290.15–303.48 K, the John–Holder model predicts that the phase equilibrium of methane hydrate will perform better. In the temperature range of 273.44–283.09 K, the John–Holder model uses the Peng–Robinson state to predict the phase equilibrium of carbon dioxide hydrate with the highest accuracy. In the temperature range of 273.68 K to 287.6 K, the Chen–Guo model is selected to predict the phase equilibrium of ethane hydrate with the highest accuracy. However, as the temperature increases, the predicted values of the vdW–P model and the Parrish–Prausnitz model deviate further from the experimental values.

## Abstract 2

Based on the 150 collected experimental data points, the accuracy of the four thermodynamic models in predicting the phase equilibrium of the gas hydrate was compared.

## Graphical abstract

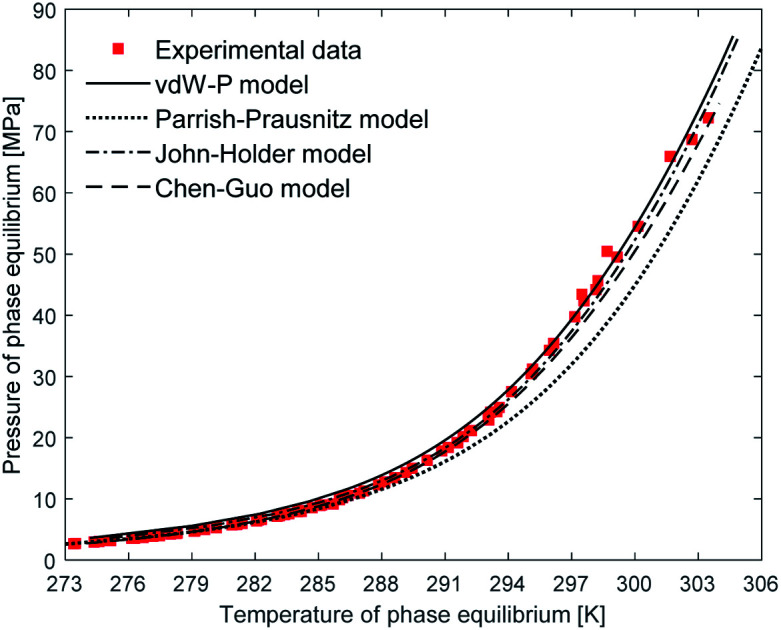

## 1. Introduction

A gas hydrate is an ice crystal-like solid formed by gas and water molecules at low temperature and high pressure.^1^ So far, more than 230 hydrate deposits have been discovered in the deep sea and polar regions.^2^ Among them, natural gas hydrate provides a type of clean energy. Meanwhile, natural gas hydrate has received considerable attention due to its essential role in energy storage.^3–5^ However, in the oil–gas field, the formed natural gas hydrates will cause device blockage and pose a serious threat to oil–gas production, transportation, and processing.^6–10^ Therefore, accurate and reliable hydrate phase equilibrium prediction is necessary for natural gas hydrate exploitation and essential for improving natural gas separation technology and preventing blockage of oil and gas pipelines.

So far, the prediction methods of phase equilibrium of gas hydrate mainly include experimental measurements, empirical models, thermodynamic models, and artificial intelligence algorithms.^11,12^ The experimental determination of hydrate equilibrium conditions requires high operating costs and is time-consuming and not conducive to engineering applications.^13^ Although it is convenient and straightforward to use empirical models to calculate the phase equilibrium of gas hydrates, the scope of application is narrow due to the excessive dependence of empirical parameters on experimental data.^14–17^ Artificial intelligence algorithms, such as neural network algorithms, are computationally complex, time-consuming, and unsuitable for engineering applications.^18–20^ In addition, the thermodynamic model is another way to predict the phase equilibrium of gas hydrates. The significant advantages of using thermodynamic models are high accuracy and wide applicable temperature range.^21–25^

The thermodynamic models that predict the formation conditions of gas hydrates are almost all based on classical statistical thermodynamics, then according to fugacity or chemical potential of the components in different phases is equal at phase equilibrium. van der Waals and Platteeuw^26^ developed a vdW–P model based on classical adsorption theory for the first time. Saito *et al.*^27^ established a method to predict the phase equilibrium of hydrate. Later, Parrish and Prausnitz^28^ generalized it. John *et al.*^29^ considered that the weakness of the vdW–P model lies in some unreasonable assumptions and made reasonable corrections to the model. In addition, the Chen–Guo model^30^ is another well-known thermodynamic model used to predict the formation condition of hydrates.

As mentioned in summary, the thermodynamic models for predicting the phase equilibrium of hydrates can be divided into two categories: the thermodynamic model based on the vdW–P model, and the other is the thermodynamic model based on the Chen–Guo model. Later, researchers revised and developed the two types of thermodynamic models. Predicting hydrate formation conditions is extended from aqueous solution to electrolyte solution and organic solvent-containing solution,^31–33^ from single gas component to multi-gas component.^34^ In addition, the temperature ranges for predicting the phase equilibrium of hydrates continues to expand.

However, the prediction results of different thermodynamic models are different. At the same time, there are differences in the accuracy of the thermodynamic model predictions in different temperature ranges. Therefore, we need to study the best applicable range of each thermodynamic model. Finally, we can use the most suitable thermodynamic model to predict the phase equilibrium conditions of natural gas hydrates in different temperature ranges.

In addition, the calculation of gas fugacity is also the key to the accuracy of model prediction results.^35^ When using the thermodynamic model for predictive analyses, we need to use the equation of state to calculate the component fugacity. For example, based on the vdW–P model, Naghibzade *et al.*^36^ used the Redlish–Kwong equations of state (RK EOS) and Patel–Teja equations of state (PT EOS) to predict the formation condition of carbon dioxide hydrate; Pang *et al.*^37^ used The Peng–Robinson equation of state (PR EOS) calculates the fugacity of the mixed gas. Based on the Chen–Guo model, Joshi *et al.*^38^ used the Soave–Redlich–Kwong equation of state (SRK EOS) to calculate the gas fugacity and obtained the phase equilibrium pressure of methane hydrate in different concentrations of tetrabutylammonium bromide solution; Barmavath *et al.*^39^ used the PT EOS to calculate the gas fugacity and got the phase equilibrium temperature of methane and carbon dioxide hydrate in porous media. Through the water fugacity model, Avula *et al.*^40^ used the PR EOS to predict the phase equilibrium prediction conditions of methane and carbon dioxide hydrate in ionic solutions; Shi *et al.*^41^ used the PR EOS to predict the formation conditions of methane and carbon dioxide to form hydrates in tetrabutylammonium halide. Liu *et al.*^42^ used the Benedict–Webb–Rubin equation of state (BWRS EOS) to predict the phase equilibrium of multi-element mixed gas hydrates. Although the above research does not include all the equations of state used to calculate the fugacity, it is found that replacing the equation of state for calculating the fugacity under the same thermodynamic model will directly affect the prediction accuracy of the phase equilibrium condition of gas hydrate.

Therefore, based on the thermodynamic model, this paper fully considers the water activity change caused by gas dissolution and compares and analyzes the prediction accuracy of different thermodynamic models in different temperature ranges. Meanwhile, RK EOS, SRK EOS, PR EOS, PT EOS, and BWRS EOS are used to calculate the gas phase fugacity to predict the phase equilibrium of the three gas hydrates of methane, ethane, and carbon dioxide. Furthermore, optimal state equations applicable to different thermodynamic models are optimized.

## 2. Thermodynamic model

The establishment of the thermodynamic model based on the classical adsorption theory is based on the equilibrium condition that the chemical potential of water in the hydrate phase and the water-rich phase is equal.

1 *μ*^H^ = *μ*^W^

where, *μ*^H^ is the chemical potential of water in the hydrate phase, and *μ*^W^ is the chemical potential of water in the water-rich phase or ice phase.

Suppose the chemical potential (*μ*^β^) of an empty hydrate phase (a hypothetical state where water molecules do not occupy the cavities of the crystal lattice) is used as a reference. In this case, the equilibrium conditions can be expressed as follows:2
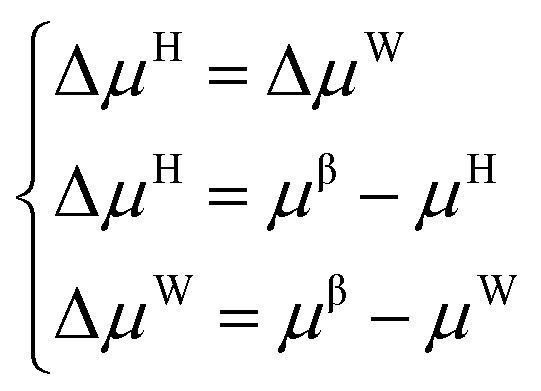
where Δ*μ*^H^ and Δ*μ*^W^ are the chemical potential deviation. According to the different calculation methods of Δ*μ*^H^ and Δ*μ*^W^, a variety of thermodynamic models for phase equilibrium prediction of gas hydrate have been developed.

### 2.1 Calculation of the Δ*μ*^H^

In order to link the Δ*μ*^H^ with the observable quantity, van der Waals and Platteeuw^26^ proposed the following hypothesis:

(1) Each cavity can only hold one gas molecule at most.

(2) The cavities are considered to be spherical, and the intermolecular potential energy function can describe the interaction between gas molecules and water molecules on the crystal lattice.

(3) The gas molecules can rotate freely in the cavity.

(4) There is no interaction between gas molecules in different cavities, and gas molecules only interact with the nearest water molecules.

(5) The contribution of water molecules to the free energy of hydrates has nothing to do with the size and type of gas molecules it contains (gas molecules cannot deform the hydrate lattice).

Based on the above assumptions, the following expression of Δ*μ*^H^ can be derived:3
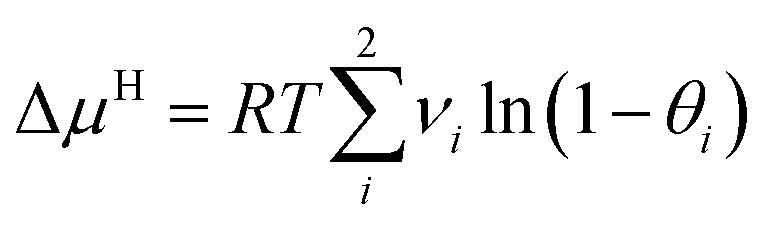
where, *ν*_*i*_ is the number of *i*-type pores per water molecule, and *θ*_*i*_ is the ratio of *i*-type pores occupied by guest molecules, as follows:4
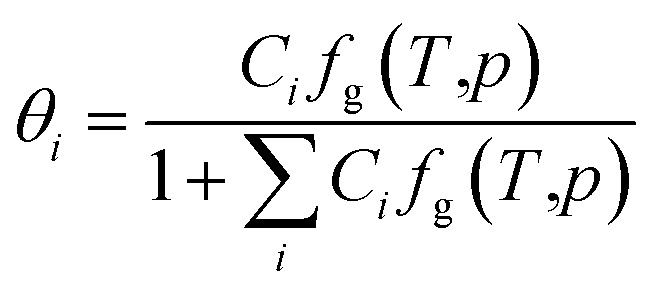
where, *C*_*i*_ is the Langmuir gas adsorption constant of guest molecules in *i*-type cavities; *f*_g_(*T*,*p*) is the gas fugacity at temperature *T* and pressure *p*.

Given the different calculation methods of *C*_*i*_, a series of thermodynamic models of phase equilibrium of gas hydrate have been developed, such as vdW–P model, Parrish–Prausnitz and John–Holder model.

#### 2.1.1. vdW–P model

The *C*_*i*_ describes the potential interaction between the encapsulated guest molecule and the surrounding water molecules in each cage. van der Waals and Platteeuw are calculated by assuming a spherically symmetric potential, as follows:5
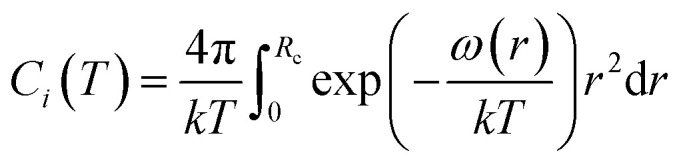
where *k* is the Boltzmann constant (*k* = 1.38062 × 10^−23^ J K^−1^); *R*_c_ is the radius of the hole; *ω*(*r*) is the sum of the potential energy between the guest molecules in the hydrate lattice cavity and the water molecules constituting the cavity.

The calculation of *ω*(*r*) depends on the molecular potential energy model used. By comparing the calculation results of several molecular potential energy models, McKoy *et al.*^43^ found that the Kihara potential energy model is better for dealing with hydrate problems. The *ω*(*r*) derived from Kihara's potential energy model is expressed as follows:6
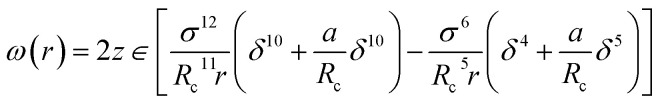
where *z* is the coordination number (the number of water molecules outside each pore); *a* is the radius of the molecular core, Å; *∈* is the energy parameter, *J*; *σ* is the distance between the molecular nuclei when the potential energy is zero, Å.

The Kihara potential energy parameters of some gases are shown in Table 1,^44^ and the calculation equation of *σ* is as follows:7
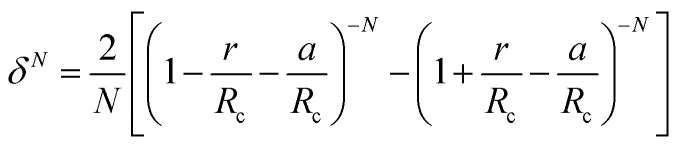
where *N* is the exponent, taking the values 4, 5, 10, or 11, respectively.

**Table 1:** Kihara potential energy parameters of gas

Gas	*a* (Å)	*σ* (Å)	*∈*/*k* (Å)
CH_4_	0.3834	3.1650	154.54
C_2_H_6_	0.6760	3.1383	190.80
C_3_H_8_	0.8340	3.1440	194.55
N_2_	0.5290	0.2569	150.03
H_2_S	0.4920	3.1774	198.53
CO_2_	0.17730	2.9605	170.97

#### 2.1.2. Parrish–Prausnitz model

Parrish and Prausnitz^28^ obtained the empirical equation for *C*_*i*_ through regression experimental data, as shown below:8
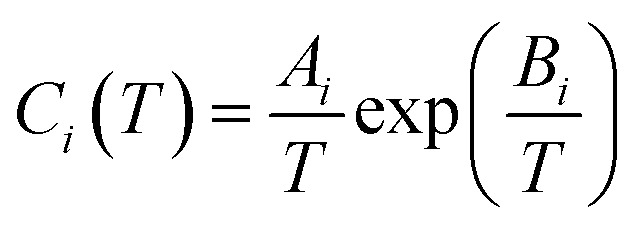
where *A*_*i*_ and *B*_*i*_ are empirical parameters, as shown in Table 2.

**Table 2:** Empirical constants for calculating *C*_*i*_

Gas	Structure I				Structure II			
	Small cavity		Large cavity		Small cavity		Large cavity	
	*A*_*i*_ × 10^3^	*B*_*i*_ × 10^−3^	*A*_*i*_ × 10^2^	*B*_*i*_ × 10^−3^	*A*_*i*_ × 10^3^	*B*_*i*_ × 10^−3^	*A*_*i*_ × 10^2^	*B*_*i*_ × 10^−3^
CH_4_	3.7237	2.7088	1.8372	2.7379	2.9560	2.6951	7.6068	2.2027
C_2_H_4_	0.0830	2.3969	0.5448	3.6638	0.0641	2.0425	3.4940	3.1071
C_2_H_6_	0.0000	0.0000	0.6906	3.6638	0.0000	0.0000	4.0818	3.0384
N_2_	3.8087	2.2055	1.8420	2.3013	3.0284	2.1750	7.5149	1.8606
H_2_S	3.0343	3.7360	1.6740	3.6109	2.3758	3.7506	7.3631	2.8541
CO_2_	1.1978	2.8605	0.8507	3.2779	0.9091	2.6954	4.8262	2.5718

#### 2.1.3. John–Holder model

John and Holder^29^ considered that the cavities are not spherical. The distances between the water molecules and the cavity centers are not equal, and used a three-layer sphere model to describe the interaction between water molecules and guest molecules. Each determines the total potential energy *W*_*i*_(*r*) of the cavities. The potential energy *W*_*i*_(*r*) of the layer shell is summed up, as shown below:

9 *W*(*r*) = *W*_1_(*r*) + *W*_2_(*r*) + *W*_3_(*r*)

where, *W*_*i*_(*r*) (*i* = 1, 2, 3) is calculated by the eqn (6) according to the characteristic structural parameters of the *i*-layer sphere, and the type parameters are shown in Table 3.

**Table 3:** Structural characteristic parameters

Structure and cavity type		First shell		Second shell		Third shell		*n*_0_	*a*_0_
		*R*_c_ (Å)	*z*	*R*_c_ (Å)	*z*	*R*_c_ (Å)	*z*		
I	Small cavity	3.875	20	6.593	20	8.056	50	0.973	35.345
	Large cavity	4.152	21	7.078	24	8.255	50	0.828	14.116
II	Small cavity	3.870	20	6.697	20	8.079	20	0.973	35.335
	Large cavity	4.703	26	7.464	28	8.782	50	2.313	782.847

To account for the influence of non-spherical molecules, John and Holder introduced a disturbance factor Q* to correct the *C*_*i*_ of spherical molecules, that is 
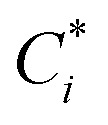
.10
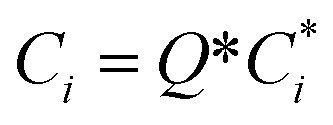
where the 
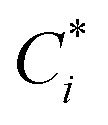
 and the Q* of the spherical molecule are calculated as follows:11
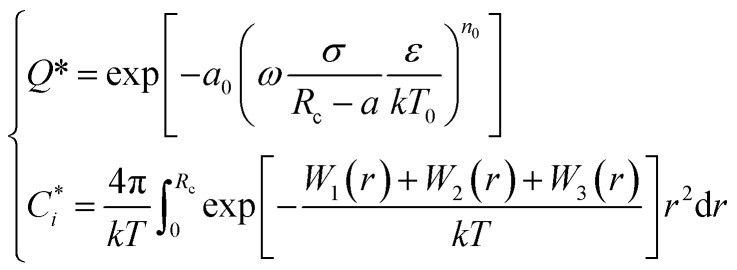
where *n*_0_ and *a*_0_ are the characteristic constants of the cavity; *ω* is the eccentricity factor of the guest molecule; *T*_0_ is the reference temperature, and the general value is 273.15 K.

#### 2.1.4. Chen–Guo model

Unlike the vdW–P model Parrish–Prausnitz model and John–Holder model, the Chen–Guo model assumes the two steps formation of gas hydrates: (1) the dissolved gas molecules in water and water molecules interact with each other to form unstable clusters. (2) The gas continues to dissolve into the water and enter the connecting cavities so that the hydrate formed no longer has stoichiometric properties.

Based on the above assumptions, Chen and Guo^30^ used statistical thermodynamics to derive the fugacity equation of guest molecules in the hydrate phase based on the kinetic mechanism of hydrate formation, as shown below:12
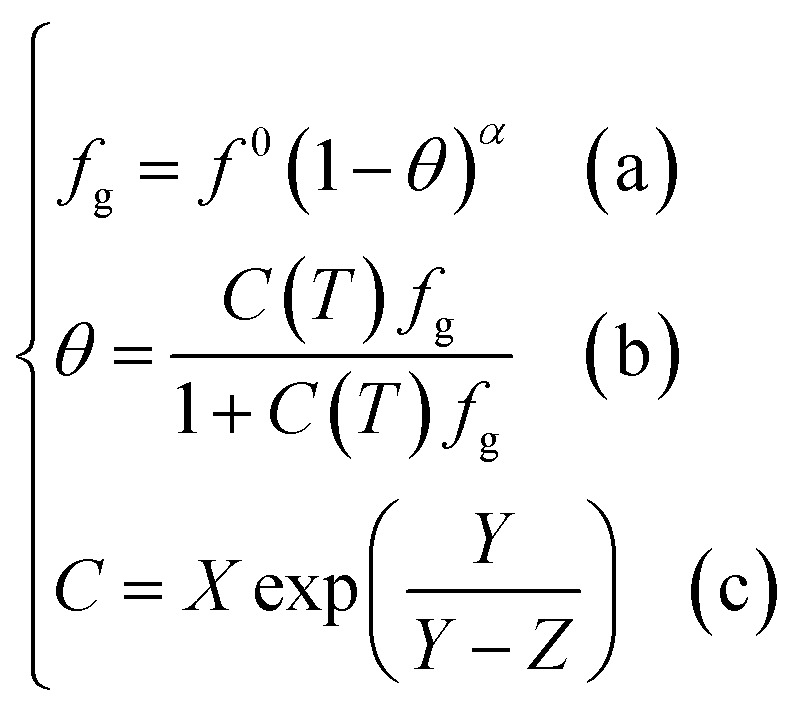
where *α* = *λ*_1_/*λ*_2_. *λ*_1_ is the number of connection cavities contained in each water molecule. When the formed hydrate is the structure I hydrate, *α* = 1/3; when it is the structure II hydrate, *α* = 1/2. *λ*_2_ is the number of gas molecules enclosed by each water molecule in the basic hydrate. For structure I hydrate, *λ*_2_ = 3/23; and for structure II hydrate, *λ*_2_ = 1/17. *f*^0^_g_ is the gas phase fugacity of basic unfilled hydrate (*θ* = 0) at equilibrium, and which is affected by temperature (*T*) pressure (*P*), and water activity (*a*_W_) and can be expressed as the product of these three factors, namely

13 *f*^0^_g_ = *f*^0^(*T*)*f*^0^(*P*)*f*^0^(*a*_W_)

where, *f*^0^(*P*) is calculated by the following equation:14
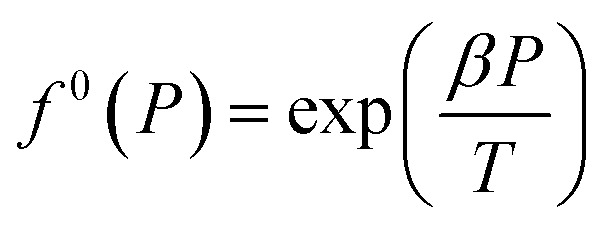
where *β* can be regarded as a constant. For structure I hydrate, *β* = 0.4242 K bar^−1^; and for structure II hydrate, *β* = 1.0224 K bar^−1^.

And *f*^0^(*a*_W_) can be obtained by the following equation:15
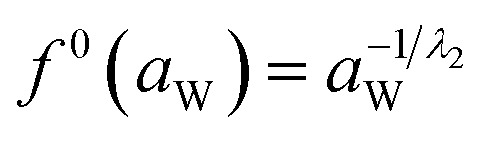


Meanwhile, *f*^0^(*T*) as a function of temperature can be obtained by Antoine equations.16
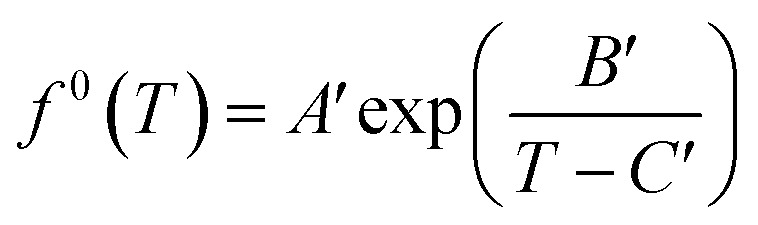


Since most pure gases only form one hydrate structure, the Antoine constants *A*′, *B*′ and *C*′ can be obtained by calculating the formation data of a pure gas hydrate with a particular structure. The Antoine constants of several typical gases are shown in Table 4.

**Table 4:** Antoine constants *A*′, *B*′ and *C*′

Gas	Structure I			Structure II		
	*A*′ × 10^15^/Pa	*B*′/K	*C*′/K	*A*′ × 10^−28^/Pa	*B*′/K	*C*′/K
CO_2_	963.72	−6444.50	36.67	3.45	−12570	6.79
H_2_S	4434.20	−7540.62	31.88	3.28	−13523	6.70
CH_4_	1584.40	−6591.43	27.04	5.26	−12955	4.08
C_2_H_4_	48.42	−5597.59	51.80	0.04	−13841	0.55
C_2_H_6_	47.50	−5465.60	57.93	0.04	−11491	30.40
C_3_H_6_	0.95	−3732.47	113.60	2.39	−13968	8.78

### 2.2 Calculation of the Δ*μ*^W^

For the pure water phase (liquid water or ice), the model proposed by Saito *et al.*^27^ to calculate Δ*μ*^W^, that is:17
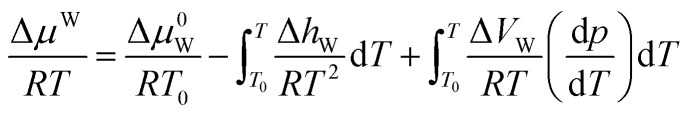
where Δ*h*_W_ is the molar enthalpy difference of water between the completely empty hydrate lattice and the pure water phase; Δ*V*_W_ is the molar volume difference between them; Δ*μ*^0^_W_ is the potential chemical difference between an empty hydrate lattice and ice under *T*_0_ (usually 273.15 K) and no pressure conditions.

For the water-rich liquid phase containing hydrocarbon solutes, Holder *et al.*^45^ assumed that Δ*V*_W_ is independent of temperature and simplified the above equation:18
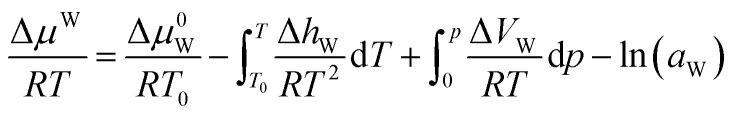
where *a*_W_ is the water activity in the water-rich liquid phase, and the water activity in the system without inhibitor is approximately equal to pure water. When the temperature is lower than the freezing point, *a*_W_ = 1; when the temperature is higher than the freezing point, it needs to be calculated according to the solubility (*x*_g_) of the gas in water. Here, we adopt the solubility calculation equation recommended by Kuustraa *et al.*,^46^ which is shown below:19
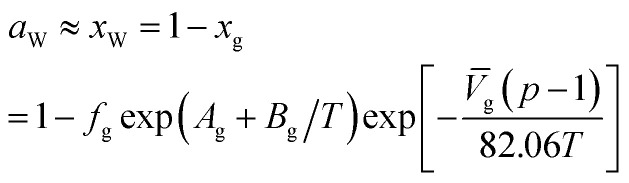
where, *x*_g_ is the solubility of the gas in the water-rich liquid phase; *V̄*_g_ is the partial molar volume of gas molecules in the water-rich liquid phase, 60 for ethylene and 32 for other gas components; *A*_g_ and *B*_g_ are model constants. The empirical values of *A*_g_ and *B*_g_ for several typical gases are shown in Table 5.

**Table 5:** Empirical constants *A*_g_ and *B*_g_

Gas	*A*_g_	*B*_g_
N_2_	−17.9343	1933.3810
O_2_	−17.1626	1914.1440
H_2_S	−15.1035	2603.9795
CO_2_	−14.2831	2050.3267
CH_4_	−15.8262	1559.0631
C_2_H_4_	−18.0579	2626.6108
C_2_H_6_	−18.4004	2410.4807

According to the thermodynamic equation, the molar enthalpy difference (Δ*h*_W_) between the completely empty hydrate lattice and the pure water phase can be expressed as follows:20
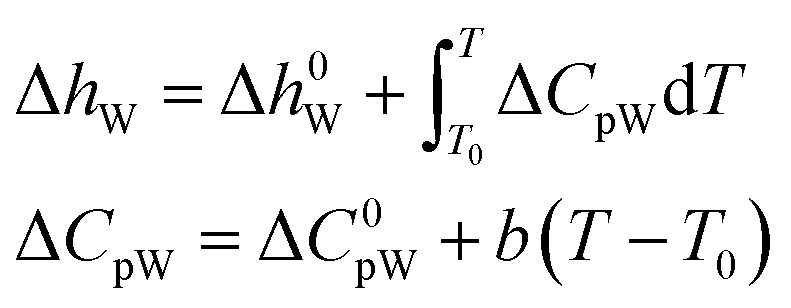
where, Δ*h*^0^_W_ is the molar enthalpy difference between water in an empty hydrate lattice and pure water phase when *T* = *T*_0_; Δ*C*^0^_pW_ is the heat capacity difference between an empty hydrate lattice and pure water phase when *T* = *T*_0_; *b* is the temperature coefficient of the specific heat capacity.

Δ*μ*^0^_W_, Δ*h*^0^_W_, Δ*C*^0^_pW_, Δ*V*_W_, and *b* must be obtained by regression of experimental data. The empirical values of those parameters of the two hydrates are shown in Table 6.^47^

**Table 6:** Regression constants Δ*μ*^0^_W_, Δ*h*^0^_W_, Δ*C*^0^_pW_, Δ*V*_W_, and *b*

Parameter	Structure I	Structure II
Δ*μ*^0^_W_ (J mol^−1^)	1120	931
Δ*h*^0^_W_ (J mol^−1^)	1714 (*T* < *T*_0_)	1400 (*T* < *T*_0_)
	−4297 (*T* > *T*_0_)	−4611 (*T* > *T*_0_)
Δ*V*_W_ (mL mol^−1^)	2.9959 (*T* < *T*_0_	3.39644 (*T* < *T*_0_)
	4.5959 (*T* > *T*_0_)	4.99644 (*T* > *T*_0_)
Δ*C*_pW_ (J mol^−1^ K^−1^)	3.315 + 0.012(*T − T*_0_) (*T* < *T*_0_)	1.029 + 0.004(*T − T*_0_) (*T* < *T*_0_)
	−34.583 + 0.189(*T − T*_0_) (*T* > *T*_0_)	−36.861 + 0.181(*T − T*_0_) (*T* > *T*_0_)

Combining the above calculation methods of Δ*μ*^H^ and Δ*μ*^W^, a set of phase equilibrium thermodynamic models of gas hydrate can be formed. The proposed model can be used to make accurate predictions of formation conditions of gas hydrate. Furthermore, by considering only the effect of inhibitor concentration on water activity, the model can be extended to predict phase equilibria under impure conditions. However, the focus of this paper is to evaluate the optimal range of applicability of various thermodynamic models and to obtain the equation of state that best matches the thermodynamic model. Therefore, we will publish the phase equilibrium prediction model under impure conditions in a subsequent study.

## 3. Model solution

The vdW–P model, Parrish–Prausnitz model, and John–Holder model are all the thermodynamic models for predicting the phase equilibrium of gas hydrate. However, the method used to obtain the Langmuir constant is different. The vdW–P model and John–Holder model determine the Langmuir constant through the potential energy function, while the Parrish–Prausnitz model uses an empirical equation to calculate it. Therefore, the solving steps of the vdW–P model, Parrish–Prausnitz model, and John–Holder model are the same as the flowchart, that is, they are all solved by eqn (2), (3) and (18).

**Fig. 1:**
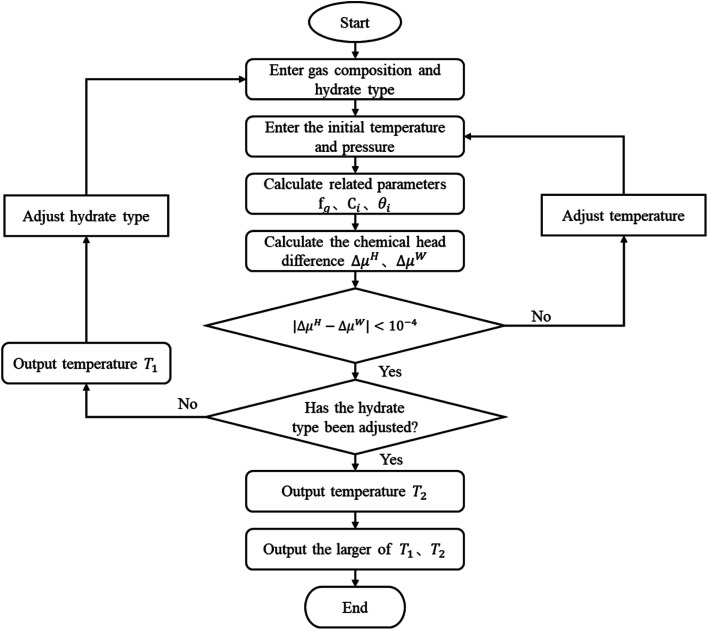
Calculation flow diagram of the vdW–P model, Parrish–Prausnitz model, and John–Holder model.

In eqn (3), there are two key parameters that need to be taken. One of is the fugacity of the gas phase, which can be calculated by RK EOS, SRK EOS, PR EOS, PT EOS or BWRS EOS, see Appendix A1. The other is the Langmuir constant of gas, which can be calculated using eqn (5), (8) or (10). The specific calculation steps are as follows, and the solution flow is shown in Fig. 1.

(1) Assuming the type of hydrate, and input the gas phase composition and formation pressure.

(2) Assign an initial value to the generated temperature (*T*_g_).

(3) Calculate the fugacity of the gas phase using the equation of state according to the gas phase composition.

(4) Find the Langmuir constant.

(5) Calculate *θ*_*i*_ according to the type of hydrate.

(6) calculate the Δ*μ*^H^ and the Δ*μ*^W^.

(7) Judge whether the |Δ*μ*^H^ − Δ*μ*^W^| is less than the set accuracy (here, we set it to 10^−4^), if this condition is met, the *T*_g_ is the phase equilibrium temperature of the type of hydrate, then return to the step (1) to change the hydrate type and calculate again. Otherwise, return to the step (2) to adjust the *T*_g_ and recalculate.

(8) Compare the temperature of the two types of formed hydrates, take the higher one as the phase equilibrium temperature of the hydrates, and the corresponding type is the type of hydrates.

**Fig. 2:**
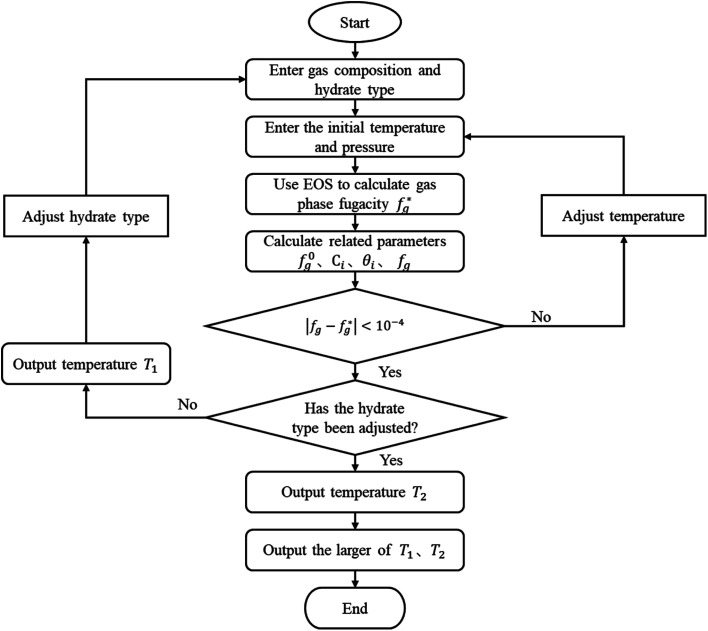
Calculation flow diagram of the Chen–Guo model.

Unlike the vdW–P model, the Chen–Guo model is solved by eqn (12) and the equation of state for calculating gas fugacity. The calculation steps of the Chen–Guo model under given pressure conditions are as follows, which calculation process is shown in Fig. 2.

(1) Assuming the type of hydrate, and input the gas phase composition and formation pressure.

(2) Assign an initial value to the generated temperature.

(3) Calculate the 
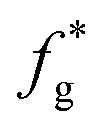
.

(4) Calculate the *f*^0^_g_.

(5) Calculate the *C* using eqn (12c), then calculate the *θ* using eqn (12b);

(6) Calculate the *f*_g_ by eqn (12a).

(7) Judge whether the 
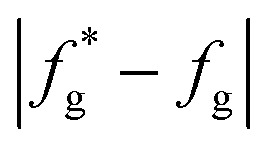
 is less than the set accuracy, if the set accuracy (10^−4^) is not met, repeat steps (2)–(6) until the requirements are met.

(8) Adjust the hydrate type and recalculate. Then compare the temperature of the two types of formed hydrates, take the higher one as the phase equilibrium temperature of the hydrates, and the corresponding type is the type of hydrates.

## 4. Results and discussion

This study used four thermodynamic models to calculate the phase equilibrium of the three gas hydrates including methane hydrate, ethane hydrate, and carbon dioxide hydrate in pure water. Based on this, the applicability of different thermodynamic models in different temperature ranges is evaluated. In addition, the influence of the five state equations on the prediction accuracy of the thermodynamic model is compared and analyzed to select the most suitable state equation.

**Fig. 3:**
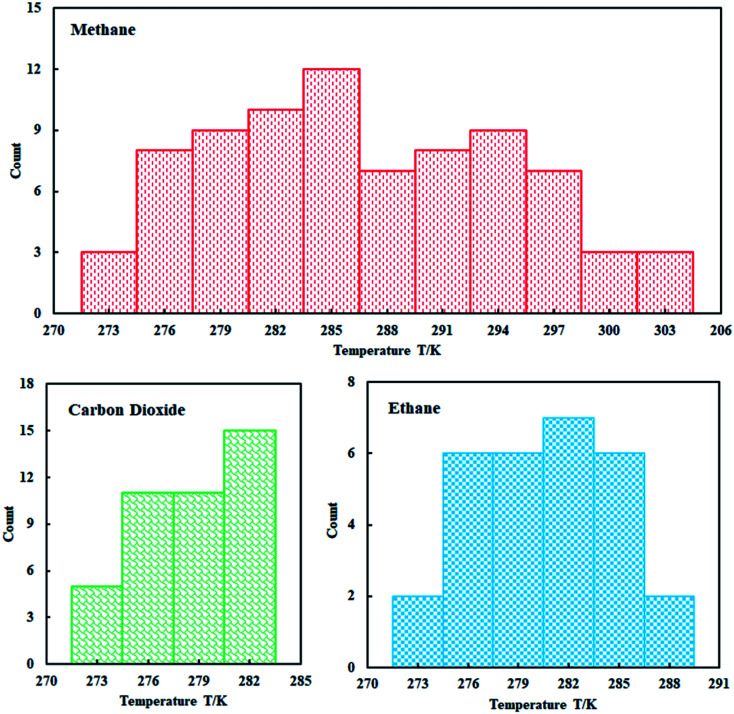
Distribution of experimental data.

A total of 150 data points for the equilibrium of hydrates in pure water were collected through research literature,^48–50^ which can be seen from Fig. 3. We can clearly understand that the collected methane hydrate phase equilibrium experimental data are concentrated between 274–303 K; ethane hydrate data are distributed between 274–288 K; and carbon dioxide hydrate data are distributed between 274–283 K.

We compared the prediction accuracy of different thermodynamic models. The evaluation standard is the average absolute deviation (AADP). The expression of AADP is:21
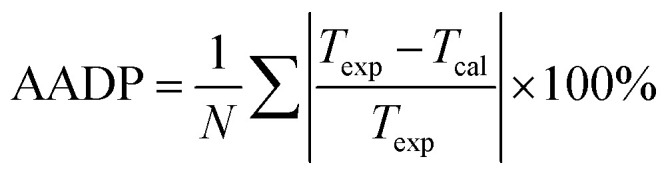


### 4.1 Comparison of prediction results of different thermal models

**Fig. 4:**
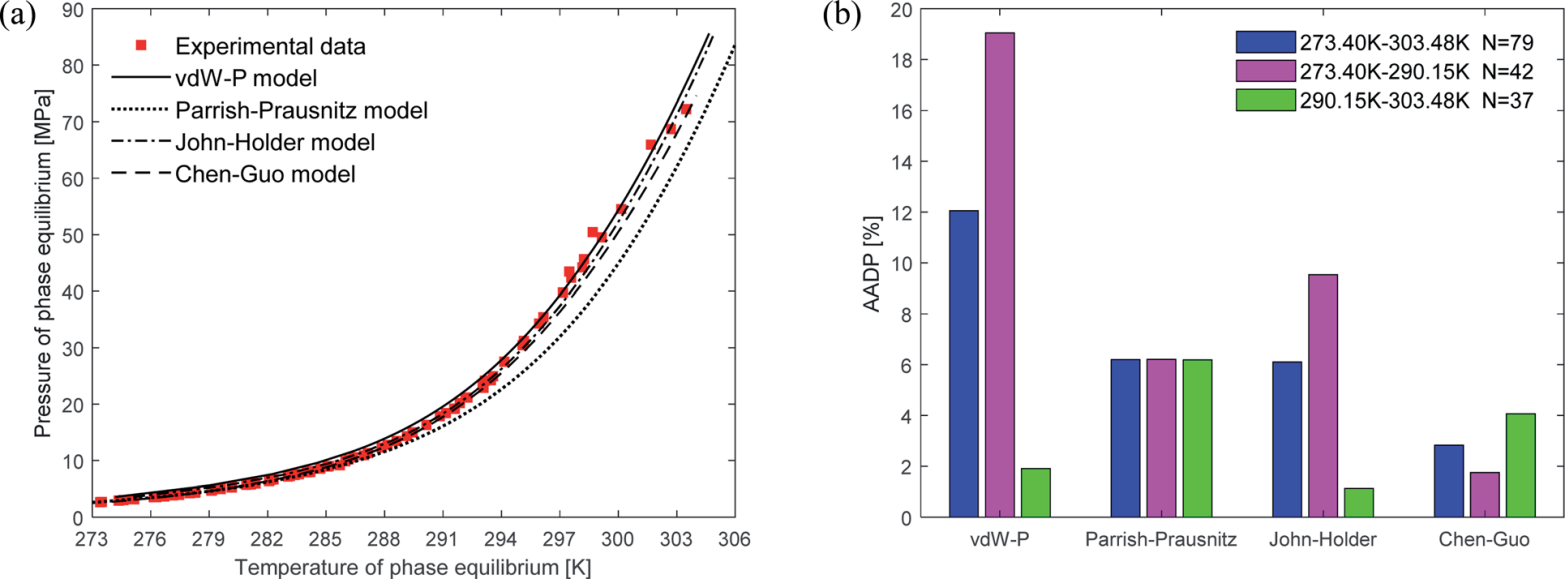
Comparison of prediction results of phase equilibrium of methane hydrate under different thermodynamic model conditions. (a) Comparison of prediction results of phase equilibrium. (b) Average absolute deviation of different thermodynamic models.

Fig. 4(a) shows the comparison curve between the predicted and experimental value of methane hydrate phase equilibrium by different thermodynamic models. The four thermodynamic models are unified using SRK EOS to calculate the fugacity of gas phase. It can be seen from the figure that the trend of the phase equilibrium curve of methane hydrate predicted by the four thermodynamic models are the same. As the temperature increases, the prediction result of the Parrish–Prausnitz model is gradually lower than the experimental value. Only the John–Holder model and Chen–Guo model are relatively close to the experimental value. In addition, due to the influence of the non-spherical cavity when the John–Holder model calculates the potential energy, the prediction result of the John–Holder model is closer to the experimental value than the vdW–P model.

Fig. 4(b) describes the statistical prediction error of each thermodynamic model. It is easy to find from the figure that the AADP of each thermodynamic model in the broad temperature range of 273.40–303.48 K is 12.04%, 6.19%, 6.10%, 2.83%, respectively. The Chen–Guo model is better than other thermodynamic models in predicting the equilibrium temperature of CH_4_ hydrate in this temperature range. However, in the temperature range of 290.15–303.48 K, the predicted accuracy of the Chen–Guo model (4.05%) is lower than the John–Holder model (1.13%). Which indicates that choosing a suitable thermodynamic model in different temperature ranges is very meaningful to improve the prediction accuracy of phase equilibrium of methane hydrate.

**Fig. 5:**
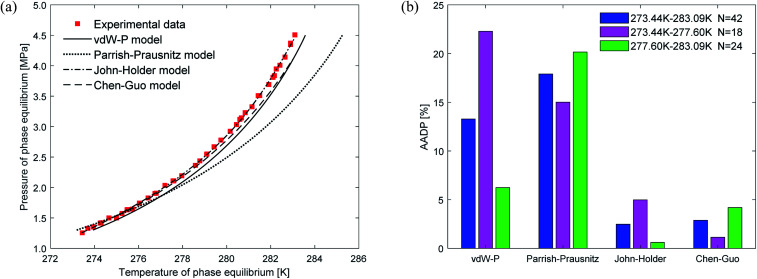
Comparison of prediction results of phase equilibrium of carbon dioxide hydrate under different thermodynamic model conditions. (a) Comparison of prediction results of phase equilibrium. (b) Average absolute deviation of different thermodynamic models.

Fig. 5(a) depicts the comparison between the predicted values and experimental values of carbon dioxide hydrate phase equilibrium under different thermodynamic model conditions. It can be seen from the figure that the predicted values of the vdW–P model and the Parrish–Prausnitz model are lower than the experimental values, while the John–Holder model and the Chen–Guo model are relatively close. In particular, as the temperature increases, there is a considerable deviation between the predicted value of the Parrish–Prausnitz model and the experimental value. Which shows that the empirical equation for calculating the Langmuir constant in the Parrish–Prausnitz model under high-temperature conditions is no longer applicable. From Fig. 5(b), it is found that in the temperature range of 273.44 K to 283.09 K, the AADP for each thermal model is 6.23%, 20.16%, 0.60%, and 4.18%, respectively. Therefore, under this temperature range, the John–Holder model performances best in predicting the phase equilibrium of carbon dioxide hydrate.

**Fig. 6:**
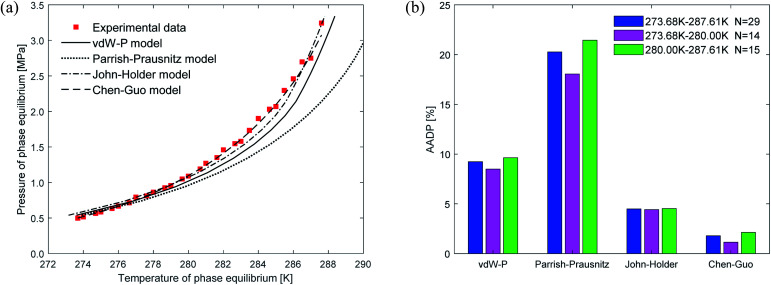
Comparison of prediction results of phase equilibrium of ethane hydrate under different thermodynamic model conditions. (a) Comparison of prediction results of phase equilibrium. (b) Average absolute deviation of different thermodynamic models.

Fig. 6 shows the prediction results of phase equilibrium of ethane hydrate in different thermodynamic models. It can be found from the figure that as the temperature increases, the predicted values of the vdW–P model and the Parrish–Prausnitz model are gradually lower than the experimental values. Only the predicted values of the John–Holder model and the Chen–Guo model are close to the experimental values. It can be seen from Fig. 6(b) that in the temperature range of 273.68–287.6 K, the AADP of the ethane hydrate phase equilibrium predicted by each thermodynamic model is 9.23%, 20.26%, 4.48%, and 1.78%, respectively. This means that the Chen–Guo model is selected to predict the phase equilibrium of ethane hydrate with the highest accuracy in this temperature range.

### 4.2 The influence of the equation of state on the predicted results

When using thermodynamic models to predict the phase equilibrium of gas hydrates, the gas phase fugacity is one of the critical parameters that affect the accuracy of the prediction results. In this study, five state equations are applied to the same thermodynamic model in turn, then the state equation most suitable for the thermodynamic model is selected. The effect of the state equation on the vdW–P model, Parrish–Prausnitz model, and John–Holder model is the same because they all have the same solution steps. Therefore, this study only uses the vdW–P model and Chen–Guo model for calculation and analysis.

**Fig. 7:**
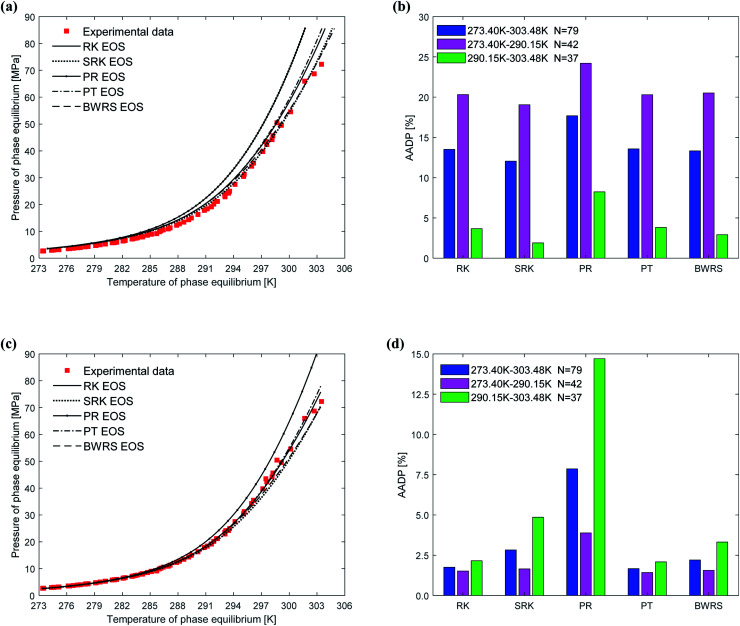
Comparison of prediction results of phase equilibrium of methane hydrate under different state equations. (a) Predicted values of vdW–P model. (b) Mean absolute deviation of the vdW–P model. (c) Predicted values of Chen–Guo model. (d) Mean absolute deviation of the Chen–Guo model.

Fig. 7 depicts the comparison between the between the predicted values and experimental values of phase equilibrium of methane hydrate in pure water under different equations of state. It's easy to find that the overall trends of the predicted phase equilibrium curves of the vdW–P model and the Chen–Guo model are the same under the five different state equation conditions. For the vdW–P model, as the temperature increases, the results predicted by the RK, PR, and PT equations of state are gradually higher than the experimental values. Among them, the results predicted by the PR equation of state have the highest degree of deviation. The BWRS expected result is the closest to the experimental value. Which can be shown in Fig. 7(a). It can be seen from Fig. 7(b) that the AADP predicted by the five equations of state in the vdW–P model under the temperature range of 273.40–303.48 K is 13.52%, 12.04%, 17.69%, 13.57%, and 13.32%, respectively.

As shown in Fig. 7(c), as the temperature rises, the results predicted by the Chen–Guo model using the PR EOS are gradually higher than the experimental value, and the results predicted by the SRK EOS and BWRS EOS are progressively lower than the experimental value. In contrast, the results predicted by the RK EOS and PT EOS are relatively close to the experimental values. When the temperature is higher than 300 K, the result predicted by the PT is closer to the experimental value than the result predicted by the RK. It can be seen from Fig. 7(d) that the AADP of Chen–Guo model uses the five state equations is 1.75%, 2.83%, 7.86%, 1.67%, and 2.21%, respectively. This means that the Chen–Guo model uses the PT EOS to predict phase equilibrium of methane hydrate with the highest accuracy in the broad temperature range of 273.40–303.48 K. At the same time, this shows that the choice of the state equation greatly influences the prediction accuracy of the thermodynamic model, too.

**Fig. 8:**
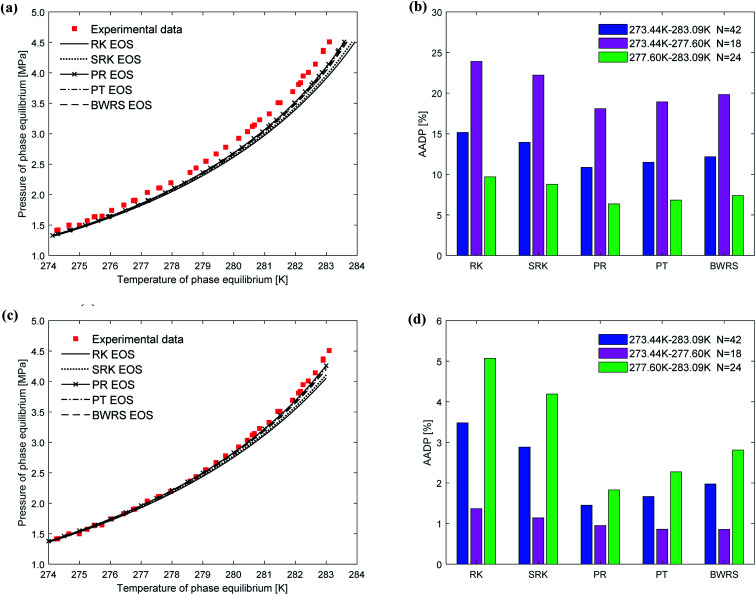
Comparison of prediction results of phase equilibrium of carbon dioxide hydrate under different state equations. (a) Predicted values of vdW–P model. (b) Mean absolute deviation of the vdW–P model. (c) Predicted values of Chen–Guo model. (d) Mean absolute deviation of the Chen–Guo model.

Fig. 8 depicts the comparison between the predicted values and experimental values of phase equilibrium of carbon dioxide hydrate in pure water under different equations of state. It can be seen from Fig. 8(a) that the results predicted by the five equations of state of the vdW–P model have slight differences. With the increase of temperature, the results are all lower than the experimental values, and the results predicted by the PR is closest. It can be found from Fig. 8(b) that the AADP predicted by the five equations of state in the vdW–P model in the temperature range of 273.44–383.09 K is 15.15%, 13.94%, 10.86%, 11.48%, and 12.17%.

It can be seen from Fig. 8(c) that the phase equilibrium curves predicted by the Chen–Guo model using different equations of state coincide. Therefore, for carbon dioxide hydrate, the choice of the equation of state has little effect on the prediction accuracy of the Chen–Guo model. It's easy to find from Fig. 8(d) that the AADP corresponding to the five equations of state is 3.48%, 2.88%, 1.45%, 1.67%, and 1.97%, respectively. Therefore, in the range of 273.36–283.3 K, the Chen–Guo model uses the PR equation of state to predict the phase equilibrium of carbon dioxide hydrate with the highest accuracy.

**Fig. 9:**
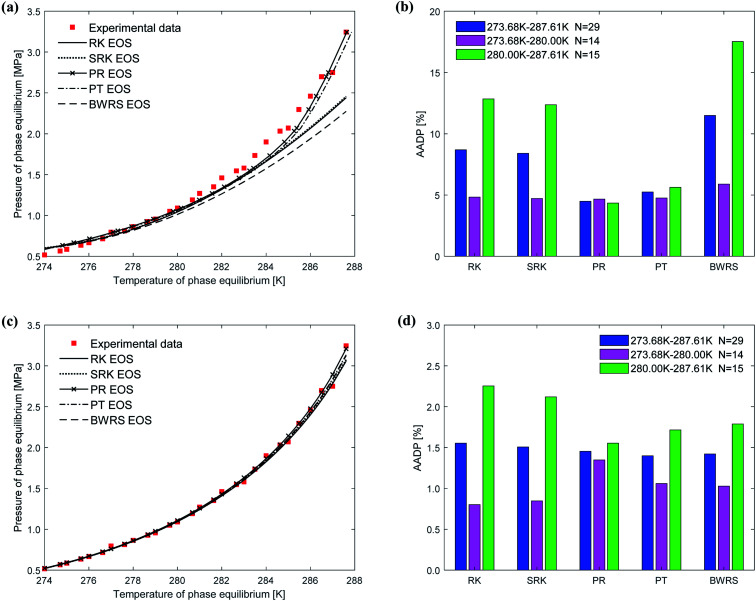
Comparison of prediction results of phase equilibrium of ethane hydrate under different state equations. (a) Predicted values of vdW–P model. (b) Mean absolute deviation of the vdW–P model. (c) Predicted values of Chen–Guo model. (d) Mean absolute deviation of the Chen–Guo model.

Fig. 9 shows the comparison between the predicted values and experimental values of the phase equilibrium of ethane hydrate under different equations of state. It can be seen from Fig. 9(a) that the prediction results of the vdW–P model using the PR EOS and PT EOS are relatively close to the experimental values. However, as the temperature increases, the prediction results of the vdW–P model using the RK, SRK, and BWRS are gradually lower than the experimental values. It can be found from Fig. 9(b) that the AADP predicted by the vdW–P model under different equations of state conditions is 8.68%, 8.40%, 4.48%, 5.24%, and 11.48%, respectively. This also shows that the choice of the state equation greatly influences the prediction results of the vdW–P model.

The phase equilibrium of ethane hydrate curves predicted by the Chen–Guo model under different equations of state coincides, which can be shown in Fig. 9(c). This shows that for ethane hydrate, the choice of the equation of state has little effect on the prediction accuracy of the Chen–Guo model. It can be seen from Fig. 9(d) that the AADP corresponding to five state equations are 1.55%, 1.50%, 1.45%, 1.39%, 1.42%, respectively. Therefore, the Chen–Guo model uses the PT equation of state to predict the phase equilibrium of ethane hydrate with the highest accuracy in the temperature range of 273.68–287.61 K.

## 5. Conclusions

In order to improve the accuracy of gas hydrate prediction, this study is based on a thermodynamic model and fully considers the changes in water activity caused by gas dissolution. The prediction results of different types of gas hydrates under various temperature ranges and different equations of state are compared. Furthermore, the thermodynamic model with the highest prediction accuracy and the corresponding equation of state is optimized. Through the verification of experimental data, this study draws the following conclusions:

(1) For CH_4_ and C_2_H_6_, the Chen–Guo model predicts better results than other thermodynamic models overall. CO_2_ and C_2_H_6_ in comparison with CH_4_, the prediction accuracy of the John–Holder model, which incorporates the effect of spherical asymmetry, is higher than that of the vdW–P model and the Parrish–Prausnitz model, referring to Fig. 4–6 in this manuscript.

(2) The higher the predicted temperature, the farther the Parrish–Prausnitz model predictions deviate from the experimental values, indicating that the empirical formula for calculating the Langmuir adsorption constants in the Parrish–Prausnitz model is no longer applicable under high temperature conditions.

(3) The vdW–P model is sensitive to the choice of state equation compared to the Chen–Guo model. The prediction results of the vdW–P model vary widely with the choice of different equations of state, especially for ethane hydrate. vdW–P model and Chen–Guo model select the PT equation of state with the highest prediction accuracy compared with other equation of state, see Fig. 7–9 in this paper.

## Appendix A

### RK EOS

The expression of the RK state equation is as follows:A-1
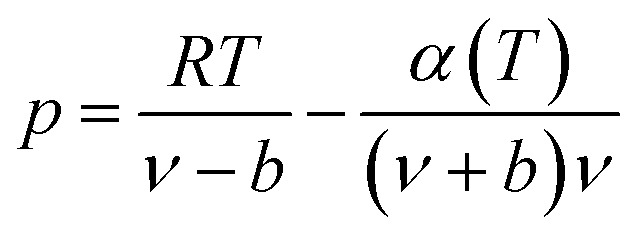


Among, *α*(*T*) = *T*^−0.5^.

When the RK EOS is used, the gas phase fugacity *f*_g_ can be expressed as:A-2
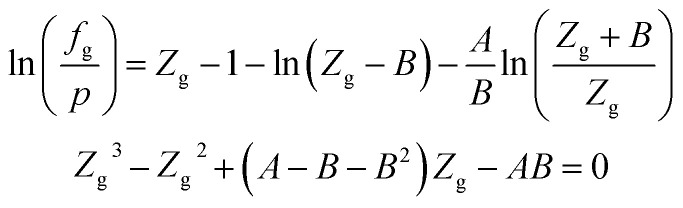


In eqn (A-2), *Z*_g_ takes the largest real root, and the expressions of *A* and *B* are:A-3
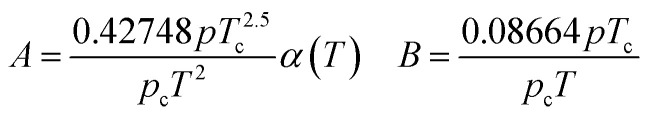


### SRK EOS

Using the SRK EOS to calculate the gas phase fugacity *f*_g_ is consistent with the RK equation of state calculation method, only the expressions of *α*(*T*) and *A* are different, namely.A-4
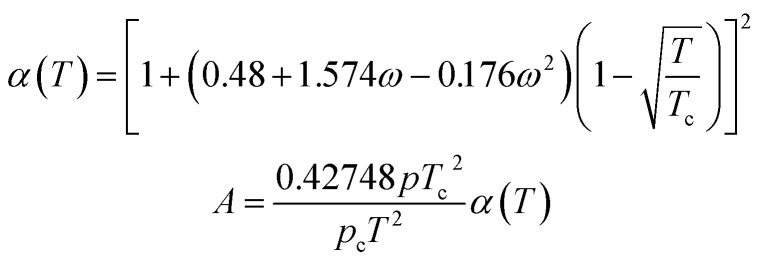


### PR EOS

The expression of the PR state equation is as follows:A-5
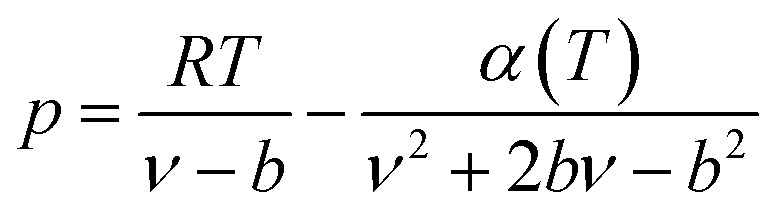


In the equation, the expression of *α*(*T*) is as follows:A-6
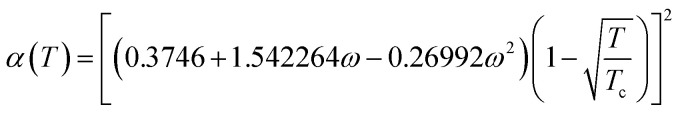


When using the PR equation of state, the gas phase fugacity *f*_g_ is expressed as follows:A-7
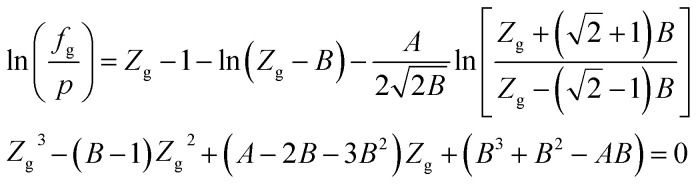


In eqn (A-7), *Z*_g_ takes the largest real root, and the expressions of *A* and *B* are:A-8
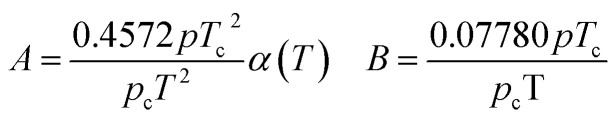


### PT EOS

The expression of the PT state equation is as follows:A-9
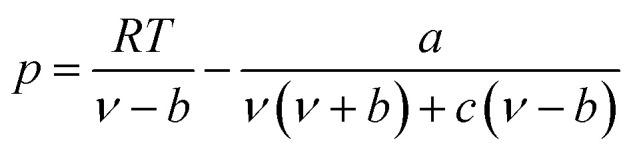


When using the PT equation of state, the gas phase fugacity *f*_g_ is expressed as follows:A-10
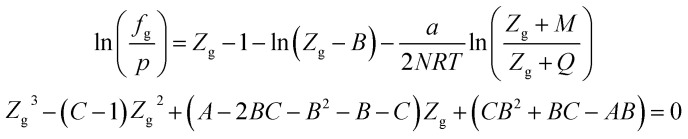


In eqn (A-7), *Z*_g_ takes the largest real root, and the expressions of *A*, *B*, *C*, *a*, *b*, *c* are:A-11
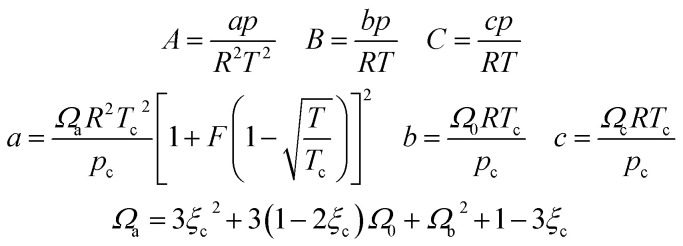


In this study, the values of *F* and *ξ*_c_ can be obtained in the literature,^51^ and the value of *Ω*_b_ is the smallest positive root of eqn (12), which is expressed as follows:A-12
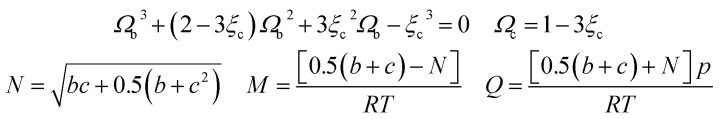


### BWRS EOS

The BWRS equation is a multi-parameter state equation, and its form is:A-13
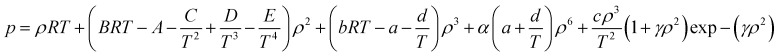


In the eqn (A-13), *A*, *B*, *C*, *D*, *E*, *a*, *b*, *c*, *d*, *α*, *γ* are the 11 parameters of the equation of state, all of which can be determined by its critical parameters *T*_c_, *p*_c_, *ρ*_c_ and eccentricity factor *ω.*A-14
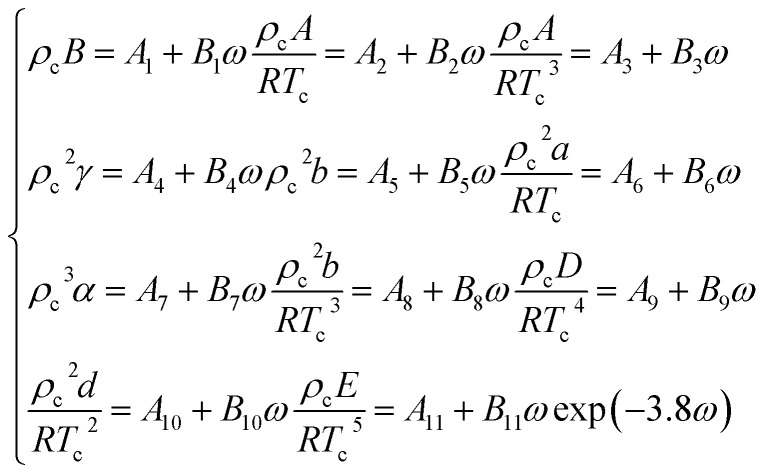


The relevant parameters in eqn (A-14) and the physical parameters of natural gas pure gas substances can be found in the literature.^52^

When using the BWRS equation of state, the gas phase fugacity *f*_g_ is expressed as follows:A-15
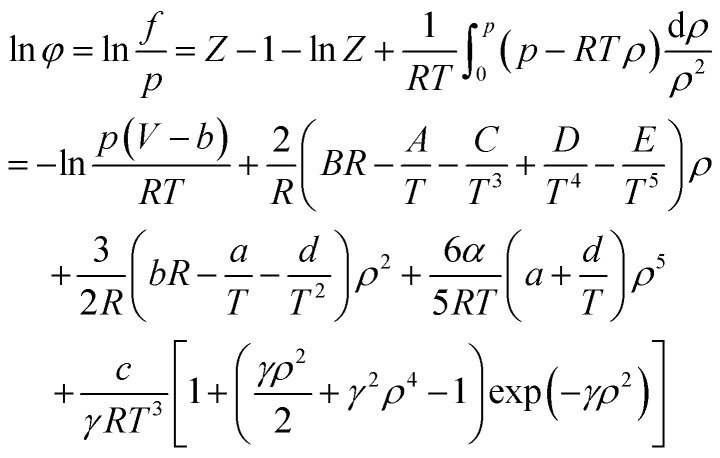


## Abbreviations

*μ* ^H^
: Chemical potential of water in hydrate phase
*μ* ^β^
: Chemical potential of empty hydrate phase
Δ*μ*^W^: Chemical potential deviation of water in water-rich phase
T
: Temperature, K
*θ*_*i*_
: Ratio of *i*-type pores occupied by guest molecules
*f*_g_
: Gas fugacity, Pa
*R*_c_
: Radius of cavity
z
: Coordination number
σ
: Distance between the molecular nuclei, Å
*n*_0_, *a*_0_: Characteristic constants of the cavity
*a*_W_
: Water activity
Δ*V*_W_: Molar volume difference
*μ* ^W^
: Chemical potential of water in water-rich phase
Δ*μ*^H^: Chemical potential deviation of water in hydrate phase
R
: Gas constant, 8.314 J (K^−1^ mol^−1^)
*ν*_*i*_
: Number of *i*-type pores per water molecule
*C*_*i*_
: Langmuir gas adsorption constant of guest molecules in *i*-type cavities
k
: Boltzmann constant, 1.38062 × 10^−23^ J K^−1^
ω
: Sum of the potential energy in the hydrate lattice cavity
a
: Radius of the molecular core, Å
Q*: Disturbance factor
*f* ^0^_g_
: Gas phase fugacity of basic unfilled hydrate
Δ*h*_W_: Molar enthalpy difference of water
*x*_g_
: Gas solubility

## Conflicts of interest

There are no conflicts to declare.

## Supplementary Material
